# Tuberculosis Outbreak in an Educational Institution in Henan Province, China

**DOI:** 10.3389/fpubh.2021.737488

**Published:** 2021-10-12

**Authors:** Hui Li, Chunfa Liu, Minghui Liang, Dongxin Liu, Bing Zhao, Jie Shi, Yanlin Zhao, Xichao Ou, Guolong Zhang

**Affiliations:** ^1^Henan Center for Disease Control and Prevention, Zhengzhou, China; ^2^National Center for Tuberculosis Control and Prevention, Chinese Center for Disease Control and Prevention, Beijing, China; ^3^Luoyang Center for Disease Control and Prevention, Luoyang, China

**Keywords:** *Mycobacterium tuberculosis*, whole genome sequencing, tuberculosis, outbreak, educational institution

## Abstract

On June 17, 2018, a case of pulmonary tuberculosis (TB) was reported among students at a senior high school in Luoning, China. The outbreak encompassed a total of 23 cases along with TB screening in the whole school by means of PPD and chest X-ray. By the end of September 2018, the entire 9 cases cultured positive had epidemiological association. All of the 9 *Mycobacterium tuberculosis* (Mtb) isolates available were sensitive to all drugs tested and had similar spoligotyping and 15 loci mycobacterial interspersed repetitive-unit-variable-number tandem-repeat (MIRU-VNTR) profile. Whole-genome sequencing (WGS) of the Mtb isolates revealed 20 variable nucleotide positions within 8 cases, indicating a clonal outbreak. The index case, which was first identified and diagnosed, is separated from the cluster by a minimum number of 95 distinct SNPs. Minimum distance spanning tree (MST) indicted that the 8 cases were indeed part of a single transmission chain. It was concluded that this is an epidemic situation of TB outbreak exposed by the aggrieved index case at school, which was caused by the veiled infectious case wherein a student was suffering from TB and attending school simultaneously.

## Introduction

China has a high prevalence of tuberculosis (TB) with strong regional disparity. In 2019, in an incidence of notified cases there were 58/100,000 population (1). TB outbreaks (cases ≥ 3) in educational institutions are occasionally reported in China (2–5). Luoning is a country in the Henan Province, which is located in central China with a 59.4/100,000 TB incidence and TB outbreak occurs occasionally in educational institution, especially in boarding schools. In 2018, 16 incidents of TB outbreak that all emerged in boarding schools in China were reported based on the public health emergencies report system. Here, we employed whole-genome sequencing (WGS) combined with field epidemiological investigation to analyze the source of a TB outbreak in Luoning, Henan.

Molecular epidemiological studies have been widely used in the phylogeography of *Mycobacterium tuberculosis* complex (MTBC) and contact tracing complemented with MTBC genotyping is considered a practical way of understanding person-to-person transmission (6, 7). Over the past decades, methods in MTBC genotyping have significantly improved. Spacer oligonucleotide typing (spoligotyping) exploits the polymorphisms of MTBC in the direct repeat (DR) region to identify and differentiate MTBC strains globally (8). MIRU-VNTR typing (mycobacterial interspersed repetitive units-variable number of tandem repeats) using a standardized set of 24 loci is currently in use for routine MTBC genotyping in many European countries and globally (9, 10). Spoligotyping and MIRU-VNTR typing are unsuitable for ruling in transmission events in China where highly conserved genotypes (mainly Beijing type) prevail.

Several studies have proved the utility of WGS in the context of epidemiological investigations following the first investigation of TB outbreak using WGS (11–14). These articles suggested that WGS provides a better discriminatory power than spoligotyping and VNTR typing to improve accuracy and to resolve false clusters (15). WGS is considered a better tool for outbreak investigations and may be able to utilize limited resources in a more targeted manner than spoligotyping and MIRU-VNTR. Mtb genome mutation rates were estimated at 0.5 single nucleotide polymorphisms (SNPs) per genome per year and a threshold of genetic distance of <6 SNP for strains from direct human to human transmission (suggesting recent transmission) was proposed (12, 15). Strain-specific SNP genotyping allowed rapid and inexpensive identification of Mtb outbreak isolated in a population-based strain study.

Here, we reported a TB outbreak among students in Luoning, with 23 cases of active pulmonary disease and 311 cases of latent infection. The index case was reported in June 2018, as a 17-year-old student in a boarding high school exhibiting symptoms such as cough, sputum, and fever since May. The index case is defined as the first case by field epidemiological investigation that was not the source of the outbreak cluster based on the SNP analysis. In conclusion, this was an epidemic situation of TB outbreak resulting from the infected student not reporting TB to the health care agent due to lack of medical examination access as well as attending school at the same time. Administration of a pre-entrance health examination to students should be considered in high-incidence countries.

## Materials and Methods

### Culture, Identification, and Drug Susceptibility Testing of Outbreak Isolates

Two sputum samples for culturing were obtained from each patient before the initiation of treatment. For isolation of the culture, each specimen was treated with one volume of 4% sodium hydroxide per one volume of sputum and was then homogenized by vigorous stirring. An aliquot of 0.1 ml of the resulting specimen was inoculated into two tubes of acidified Löwenstein–Jensen medium and incubated at 37°C. The culture was assessed during week 1 for rapidly growing bacteria and again every week thereafter for slower growing bacteria; if no bacteria grew by 8 weeks, the result was recorded as negative. Sputum samples were mainly cultured for tuberculosis at the reference laboratory of Henan CDC. Isolates were primarily identified by using X-pert MTB/RIF test (Cepheid, USA), or real-time fluorescence PCR analyzer (ZhiShan, China). Mtb complex species identification was verified by MALDI Biotyper (Bruker, Germany) at NRL, China Center for Disease Control and Prevention (CDC) (16), which receives all strains in China for identification, genotyping, and susceptibility testing when needed. Strains were tested for drug susceptibility using microtiter plates (MYCOTBI, Thermo-fish, USA).

### Spoligotyping

McSpoligotyping commercial kits (Zeesan Biotech, Xiamen, China) were used to perform spacer oligonucleotide typing (spoligotyping) analysis as described (17). Briefly, it is a one-step spoligotyping protocol based on real-time PCR to select 43 specific sequences of intervals. The result data of spoligotying were compared with SITVIT2 database (http://www.pasteur-guadeloupe.fr:8081/SITVIT2/) to obtain the spoligotype international type (SIT) number and lineage information. SpoTyping, a fast and accurate *in silico* spoligotyping of *M. tuberculosis* isolates from next generation sequencing reads, was used as showed (18). SpoTyping is freely available at: https://github.com/xiaeryu/SpoTyping-v2.0. The results obtained from two methods were compared with each other and got the same results.

### Genotyping

Routine 15-locus MIRU-VNTR testing was performed using capillary electrophoresis and commercial kits (GenoScreen, Lille, France) at NRL as described elsewhere (19). The MIRU-type was defined after combining the results for the 15 loci in the following order: 580 (MIRU4), 2996 (MIRU26), 802 (MIRU40), 960 (MIRU10), 1644 (MIRU16), 3192 (MIRU31), 424 (Mtub04), 577 (ETRC), 2165 (ETRA), 2401 (Mtub30), 3690 (Mtub39), 4156 (QUB4156), 2163b (QUB11b), 1995 (Mtub21), and 4052 (QUB26). Online analysis tool MIRU-VNTR plus (www.miru-vntrplus.org) was used to analysis the VNTR profiles.

### WGS of Mtb DNA

WGS of the 9 isolates was performed at Institute of Microbiology, Chinese Academy of Science. Briefly, DNA from the strains was extracted using the CTAB method (20, 21). DNA sequencing libraries were prepared as previously described and sequencing was performed on the Illumina MiSeq platform (Illumina, San Diego, CA), with all reads performed at 2,150 bp at a median depth of 352 (range, 52–706). The median pair distance was 221 bp (193–245 bp) and the median template coverage was 99.14–99.21%.

### Bioinformatics Analyses

Genomic DNA was subjected to WGS using the Illumina HiSeq 2500 system with the paired-end strategy as described previously. H37Rv sequence used to align the reads was NC_000962.3. SNPs located in or within 50 bp of regions annotated as PE/PPE genes, and repeat regions within 10 bp from each other, with a read depth of 8 or present in 75% of the reads in at least one isolate, were excluded from the analyses. A maximum-likelihood (ML) phylogeny and a minimum-spanning network were computed in SAM-TB (*M. tuberculosis* genomic sequence analysis platform developed in China, http://183.62.138.75/index). The ML tree was visualized and edited using MEGA7.

### Statistical Analysis

Odd ratios with 95% confidence interval (CI) were estimated and variables with *P* < 0.05 were taken as significant predictors.

## Results

### Description of Index Case and Epidemiological Investigation

On June 17, 2018, a case of childhood pulmonary TB was reported to the local CDC in Luoning with a population of ~500,000 (regional TB incidence of 57/100,000 in 2018). A 17-year-old student had been admitted to the hospital for a diagnostic workup because of a 4-week history of fever and productive cough. Prior to admission, the teenager had been diagnosed with influenza, and received therapy. On admission, the teenager underwent tuberculin skin testing (TST, Mantoux method: positive, 16 mm), a chest X-ray (CXR) (multiple patchy opacity in bilateral lung), and a presumptive diagnosis of TB was made. Later, smear microscopy came back positive and a standard treatment for TB was started. A source tracing investigation was initiated immediately, using the “progressive, concentric circles” approach (22), by screening household contacts, primary classmates, school staff, and roommates, defined as contacts with a cumulative exposure of 8 h in a small space equivalent to a domestic space (22). Screening was performed by clinical symptoms, TST, and/or CXR, according to national guidelines (5). A positive TST was defined as induration >5 mm, and CXR to exclude pulmonary active disease. Contacts with CXR abnormalities compatible with TB underwent further diagnostic evaluation, including smear microscopy and/or PCR (sputum) and culture if appropriate.

As shown in Table 1 in the first row, a total of 83 close contacts of the index case, including 66 classmates (class II-B16), 6 household contacts, 6 teachers, and 6 roommates (one also in the same class) were tested. No active TB and latent tuberculosis infection (LTBI) were found among 6 household contacts of the index case. One of the six school staff had a strongly positive TST, while CXR excluded active pulmonary TB. One of the roommates with a negative TST showed clinically productive cough. Further diagnostics found that despite the negative results of microscopy, PCR, and culture, a clinical diagnosis of TB was made after ruling out other possibilities (Table 2, case 13). A total of 15 classmates of the index case (case 5) (class II-B16) had a positive TST. Only one student with suspicious CXR findings was confirmed with pulmonary TB by culture analysis (Table 2, case 11). Another 6 students with negative TST results and suspicious CXR findings were diagnosed with active pulmonary TB (Table 2, cases 1, 6, 8, 9, 10, and 12). Among those 6 students, sputum smear microscopy and culture analysis results were positive in four cases while the other two cases were PCR-positive. To conclude, 15 contacts of the index case were found as LTBI and 8 confirmed as active pulmonary TB.

**Table 1:** Primary case contact investigations during a school tuberculosis outbreak, Luoning, Henan, China 2018.

	**Number at risk**	**Number screened**	**LTBI**			**Active**			**Infected**		
			***n***	**%**	**(95% CI)**	***n***	**%**	**(95% CI)**	***n***	**%**	**(95% CI)**
**STEP ONE**											
**Students and staff of class B16, Grade II**											
Class II-B16	66	66	14	21.21%	(11.35–31.08)	7	10.60%	(3.17–18.03)	21	31.82%	(20.58–43.06)
Roommate	6 (1)^∧^	6 (1)^∧^	0	0	(0–46)	1	16.67%	(0–64)	1	16.67%	(0–64)
Household contacts	6	6	0	0	(0–46)	0	0	(0–46)	0	0	(0–46)
School staff in class B16	6	6	1	16.67%	(0–64)	0	0	(0–46)	1	16.67%	(0–64)
*Subtotal*	*83*	*83*	*15*	*18.07%*	*(9.79–26.35)*	*8*	*9.64%*	*(3.29–15.99)*	*23*	*27.71%*	*(18.08–37.34)*
**STEP TWO**											
**School contacts in Grade II**											
School in class B	1,078	1,070	43	4.02%	(2.84–5.2)	10 (1)^#^	0.93%	(0.35–1.51)	53(1) ^#^	4.95%	(3.65–6.25)
School in class A	1,213	1,170	58	4.96%	(3.71–6.2)	4	0.34%	(0.01–0.67)	62	5.30%	(4.02–6.58)
*Subtotal*	*2,291*	*2,240*	*101*	*4.51%*	*(3.65–5.37)*	*14*	*0.63%*	*(0.3–0.96)*	*115*	*5.13%*	*(4.22–6.05)*
**STEP THREE**											
**School contacts in Grade III (Repetition class)**
School graduated 2018	308	308	8	2.60%	(0.82–4.37)	0	0	NA	8	2.60%	(0.82–4.37)
*Subtotal*	*308*	*308*	*8*	2.60%	*(0.82–4.37)*	*0*	*0*	*NA*	*8*	2.60%	(0.82–4.37)
**Staff of school**											
School staff except class B16, Grade II	524	410	12	2.93%	(1.3–4.56)	0	0	NA	12	2.93%	(1.3–4.56)
*Subtotal*	*524*	*410*	*12*	*2.93%*	*(1.3*–*4.56)*	*0*	*0*	*NA*	*12*	*2.93%*	*(1.3*–*4.56)*
**School contacts in Grade I (health examination for enrollment)**											
School enrolled 2018	2,370	2,261	175	7.74%	(6.64–8.84)	1	0.04%	NA	176	7.78%	(6.68–8.89)
*Subtotal*	*2,370*	*2,261*	*175*	7.74%	*(6.64*–*8.84)*	*1*	0.04%	*NA*	*176*	7.78%	*(6.68*–*8.89)*
**STEP FOUR**											
**Active TB cases' close contacts**											
Household contacts	90	83	0	0	NA	1	1.20%	NA	1	1.20%	NA
*Subtotal*	*90*	*83*	*0*	*0*	*NA*	*1*	1.20%	*NA*	*1*	1.20%	*NA*
**Total**	**5,666**	**5,385**	**311**	**5.78%**	**(5.15–6.4)**	**23**	**0.43%**	**(0.25–0.6)**	**334**	**6.20%**	**(5.56–6.85)**

# *One case that is in the same class and dormitory with the index case*.

**Table 2:** Individual details of the 23 cases of active pulmonary disease in Luoning High School, Henan, China, 2018.

**Case No**.	**Age**	**Sex**	**Original class**	**Class**	**Dormitory**	**Symptom**	**Discovery**	**Chest X-ray**	**PCR**	**Microscopy**	**Culture**	**Diagnosis**
1	18	F	II-B15	II-B16	4-512	Cough, sputum	Contact screening	Multiple patchy opacity in bilateral lung	Negative	Positive	Confirmed	Pulmonary Tuberculosis
2	17	M	II-B03	II-B08	1-205	Asymptomatic	Contact screening	Pleural thickening and calcification	Negative	Negative	Confirmed	Pulmonary Tuberculosis
3	17	F	II-B17	II-B18	4-512	Chest tightness	Clinical consultation	Increased opacity in left lung	Negative	Negative	Pending	Pulmonary Tuberculosis
4	19	F	II-B14	II-B13	4-405	Asymptomatic	Contact screening	Increased opacity in right upper lung	Negative	Negative	Confirmed	Pulmonary Tuberculosis
5	17	M	II-B16	II-B16	N3-408	Cough, sputum, fever	Clinical consultation	Multiple patchy opacity in bilateral lung	Negative	Positive	Confirmed	Pulmonary Tuberculosis
6	18	M	II-B13	II-B16	N3-211	Asymptomatic	Contact screening	Multiple patchy opacity	Positive	Negative	Pending	Pulmonary Tuberculosis
7	19	M	II-B17	II-B15	-	Fever	Clinical consultation	Increased opacity in right lung	Negative	Negative	Confirmed	Pulmonary Tuberculosis
8	17	F	II-B08	II-B16	4-506	Asymptomatic	Contact screening	Multiple patchy opacity	Positive	Negative	Pending	Pulmonary Tuberculosis
9	18	F	II-B15	II-B16	4-502	Asymptomatic	Contact screening	Multiple patchy opacity in right upper lung	Positive	Positive	Confirmed	Pulmonary Tuberculosis
10	17	M	II-B15	II-B16	N3-211	Cough, sputum	Contact screening	Poorly defined patchy opacity in bilateral lung	Negative	Positive	Confirmed	Pulmonary Tuberculosis
11	17	F	II-B18	II-B16	4-506	Asymptomatic	Contact screening	Multiple opacity in right upper lung	Negative	Negative	Confirmed	Pulmonary Tuberculosis
12	18	F	II-B14	II-B16	4-410	Cough	Contact screening	Cloudy opacity in right upper lung, cavitary TB	Negative	Positive	Confirmed	Pulmonary Tuberculosis
13	17	M	II-B17	II-B18	N3-408	Cough	Contact screening	Increased opacity in bilateral lung	Negative	Negative	Pending	Pulmonary Tuberculosis
14	17	M	II-B14	II-B14	N3-207	Asymptomatic	Contact screening	Increased opacity in right upper lung	Negative	Negative	Pending	Pulmonary Tuberculosis
15	17	F	II-B13	II-B14	4-410	Fever	Clinical consultation	Patchy opacity in right hilar	Negative	Negative	Pending	Pulmonary Tuberculosis
16	18	M	II-B13	II-B14	N3-207	Asymptomatic	Contact screening	Nodules in left lung	Negative	Negative	Pending	Pulmonary Tuberculosis
17	18	F	II-B09	II-B11	4-316	Cough	Contact screening	Blunt of right costal diaphragm	Negative	Negative	Pending	Tuberculous Pleurisy
18	18	F	II-B07	II-B02	4-111	Asymptomatic	Contact screening	Increased opacity in left upper lung	Positive	Negative	Pending	Pulmonary Tuberculosis
19	18	F	-	II-A18	S3-603	Asymptomatic	Contact screening	Multiple patchy opacity in right upper lung	Negative	Negative	Pending	Pulmonary Tuberculosis
20	15	F	-	II-A13	S3-403	Asymptomatic	Contact screening	Increased opacity in right upper lung	Negative	Negative	Pending	Pulmonary Tuberculosis
21	17	M	-	II-A12	1-616	Asymptomatic	Contact screening	Consolidation in superior lobe of right lung	Negative	Negative	Pending	Pulmonary Tuberculosis
22	18	M	-	II-A02	1-417	Cough	Contact screening	Increased opacity in right lower lung	Positive	Negative	Pending	Pulmonary Tuberculosis
23	17	F	I-B16	I-B16	5-616	Asymptomatic	Contact screening	Patchy opacity in right upper lung, cavitary TB	Negative	Negative	Pending	Pulmonary Tuberculosis

*Case no. 5 as the index case*.

### Expand Screening to Find More Cases

All 8 active pulmonary TB cases have mild clinical symptoms for <1 month or are asymptomatic. Despite case 12 being found to have cavitary TB, a further diagnosis reported a non-productive cough and a negative microscopy. No contacts were confirmed as the primary case, and a decision was made to expand screening of the entire Grade-II population considering their major changes to either science or liberal arts 5 months ago (Table 1, “Step two”).

The Grade-II was a five-story building and was divided into two departments (Class A and Class B). Class II-B16 was on the west side of the fourth floor with Class II-B13, 14, and 15. Nine additional active TB cases in Class B (five were on the same floor with the index case) and four in Class A were identified (Table 1). Therefore, the investigation was further extended as shown in Table 1 “Step three,” to all the school staff first, and subsequently to students who had graduated but decided to remain in Grade-III for another year (some lived in the same dormitory with students in Grade-II) and the students in Grade-I (a different building from the Grade-II building). Of the 410 school staff and 308 students in Grade-III, 12 and 8 were TST-positive (both had negative CXR), respectively. In Grade-I, 175 students were LTBI positive and 1 was active pulmonary TB with cavitary TB (Table 2, case 23). A further investigation found that case 23 had suffered with diabetes and had a history of TB exposure within the family.

Overall, as shown in Table 2, 23 cases of active TB were identified, with 13 females and 10 males. All cases were students [17 from Class II-B (8 from II-B16), 4 from Class II-A, and 1 from Grade-I]. In addition, 298 students and 13 teachers in the whole school were diagnosed with LTBI (Table 1). In all, 50 cases with strongly TST-positive (induration ≥15 mm or blisters and other reactions) and normal radiological features were advised to receive isoniazid preventive therapies against TB on a voluntary basis and 19 cases accepted.

Further investigation (Table 1, Step four) was performed to screen the contacts within the household of the 23 active pulmonary TB cases (return home 1 day every 2 weeks). None of the household contacts of these cases in Grade-II reported a TB history or a positive infective result, so the possibility of household transmission was excluded. A family member of case 23 was reported to have a TB infection in the past and had been undertaking anti-TB treatment. The screening may avoid a potential transmission by case 23 in Grade I. Table 1 shows attack rates observed in the outbreak and 32% of student attending class II-B16 were infected.

### Characteristics of Outbreak Isolates

The first patient (case 5) recognized as part of the outbreak was a student at a senior high school in Luoning, China in June 2018. Contact tracing around this case included 5,385 people, among which 23 were diagnosed with active TB. We analyzed the characteristics of the 9 strains from the cultured positive students (case 1, 2, 4, 5, 7, 9, 10, 11, and 12). All 9 strains belong to MTBC defined by MADLI-TOF-MS. Drug susceptibility testing was tested by MIC plates. All of them were susceptible to first-line TB drugs isoniazide (INH), rifampicin (RIF), and ethambutol (EMB); amikacin (AMK); and ofloxacin (OFX) (Table 3). This implied an outbreak of non-drug-resistant TB in school.

**Table 3:** Characteristics of outbreak isolates.

**Case**	**Age**	**Sex**	**Class**	**Dormitory**	**MADLI-TOF-MS**	**Antibiotic susceptibility**				
						**INH**	**RIF**	**EMB**	**AMK**	**OFX**
1	18	F	II-B16	4-512	MTBC	S	S	S	S	S
2	17	M	II-B08	1-205	MTBC	S	S	S	S	S
4	19	F	II-B13	4-405	MTBC	S	S	S	S	S
5 (index case)	17	M	II-B16	N3-408	MTBC	S	S	S	S	S
7	19	M	II-B15	-	MTBC	S	S	S	S	S
9	18	F	II-B16	4-502	MTBC	S	S	S	S	S
10	17	M	II-B16	N3-211	MTBC	S	S	S	S	S
11	17	F	II-B16	4-506	MTBC	S	S	S	S	S
12	18	F	II-B16	4-410	MTBC	S	S	S	S	S

*INH, Isoniazide; RIF, Rifampicin; EMB, Ethambutol; AMK, Amikacin; OFX, Ofloxacin*.

### Outbreak Defined by Spoligotyping and 15 Loci MIRU-VNTR

**Figure 1:**
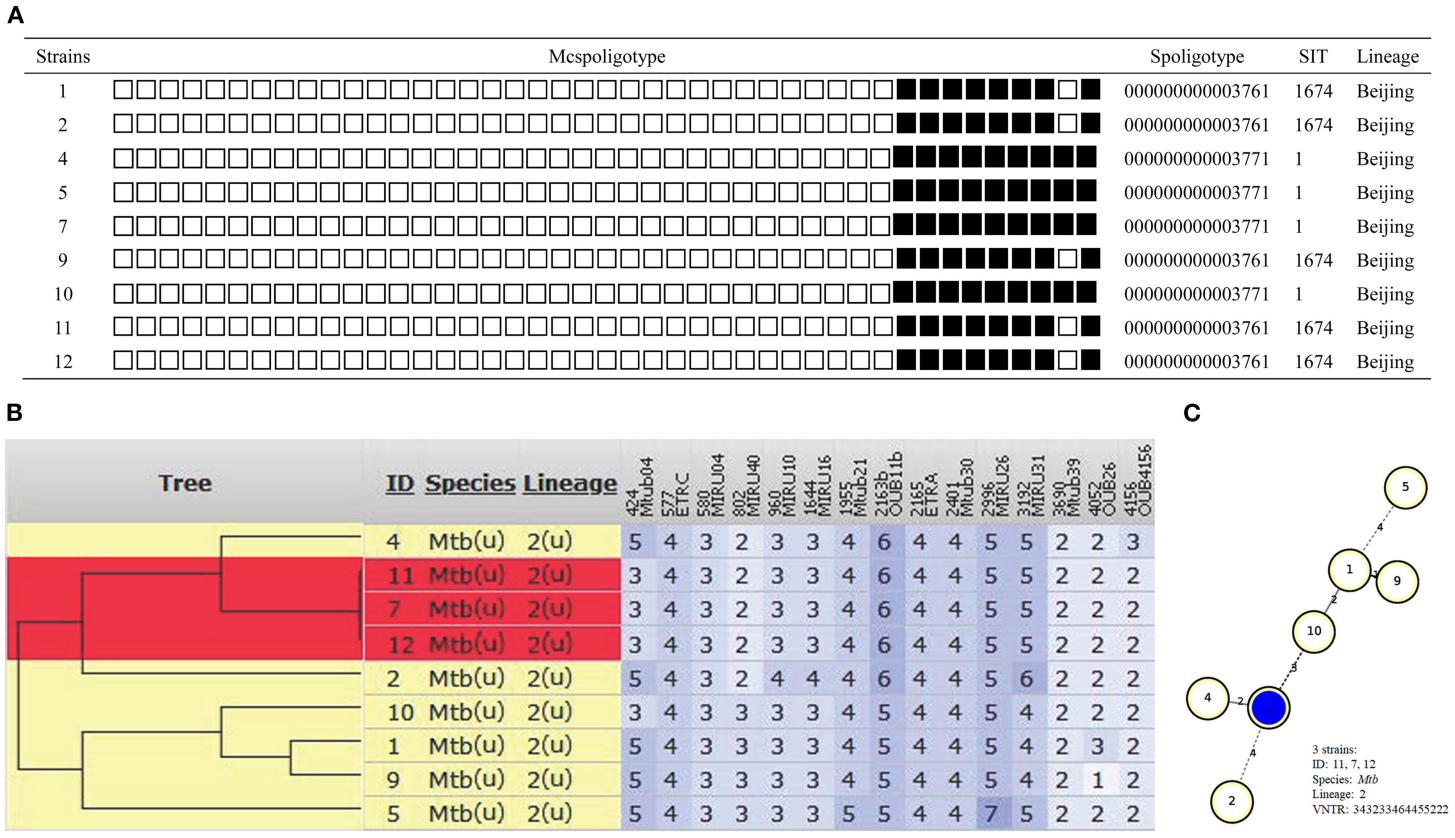
Outbreak defined by spoligotyping and 15 loci MIRU-VNTR. **(A)** Spoligotyping genotype. **(B)** The phylogenetic tree based on 15 locus MIRU-VNTR genotype. **(C)** MST based on MIRU-VNTR genotype.

Overall, all isolates shared same spoligotyping results by using SpoTyping and McSpoligotyping methods. As showed in Figure 1A, 9 strains had two genotypes (SIT1 and SIT1674) and all were Beijing type. Of the 9 cases, 3 (case 7, 11, 12) had an indistinguishable 15 locus MIRU-VNTR genotype 343233464455222. The other 6 isolates showed different genotypes (Figure 1B). MST (minimum spanning tree) based on MIRU-VNTR genotype demonstrated 0–4 distances among these 9 isolates (Figure 1C).

### WGS

**Figure 2:**
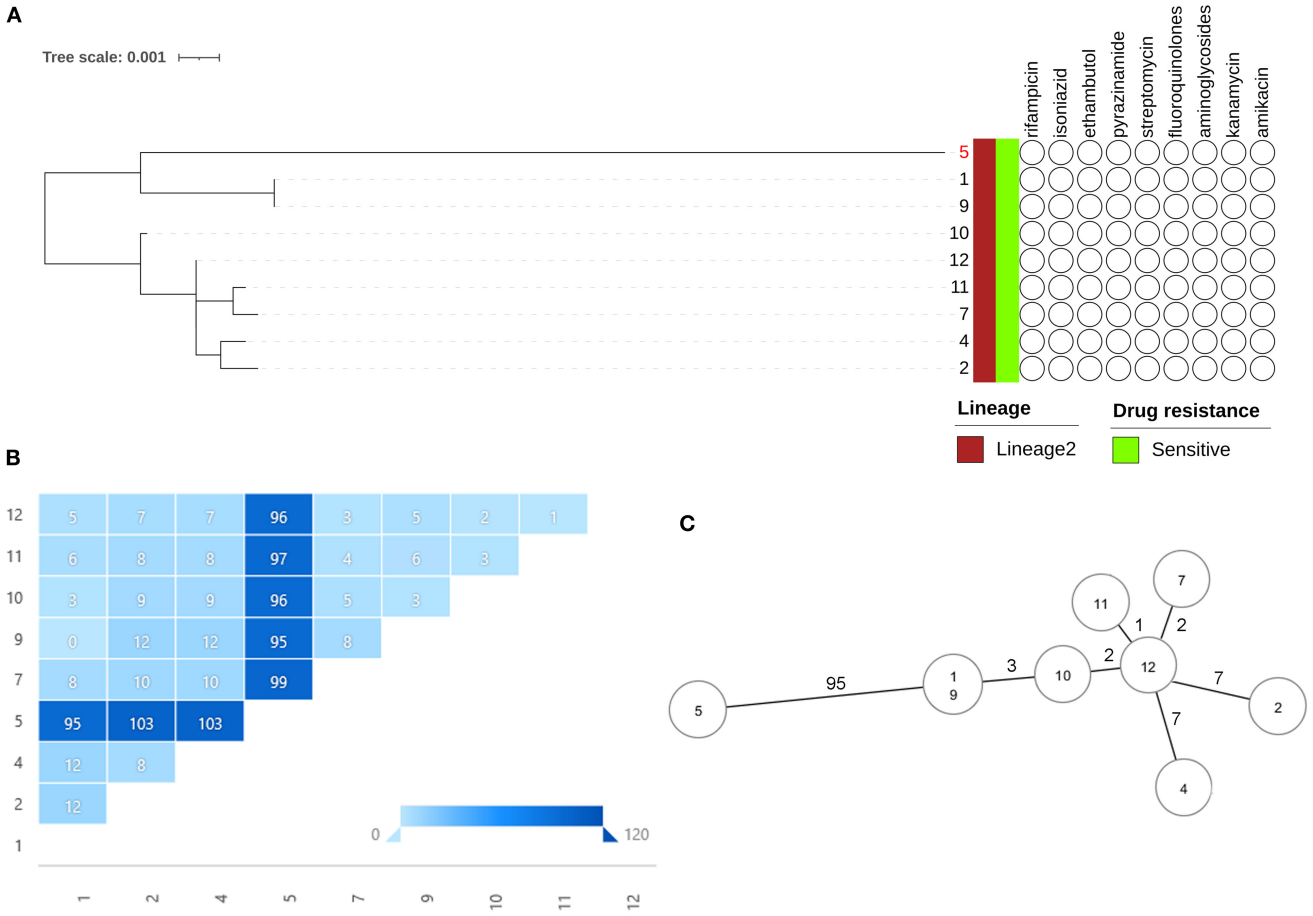
Outbreak defined by WGS. **(A)** The phylogenetic tree combination with lineage and genotypic drug resistance of Mtb isolates (index case 5 showed in red). **(B)** SNP distances between each two Mtb isolates. **(C)** MST based on the SNP distances.

As the phylogenetic tree shown in Figure 2A, all 9 strains belong to lineage 2, and no drug genotypic resistance was found. The SNP distances between each two strains were calculated (Figure 2B), all 8 strains obtained <12 SNP distances except for 1 strain with case 5. To intuitively visualize the precise population structure and spreading of the cluster isolates, we calculated an MST based on the concatenated SNP sequences (Figure 2C). The SNP-based cluster analysis distinguished the 9 outbreak isolates into 1 major cluster with 8 isolates in which the maximum SNP distance is not larger than 12 SNPs [within the range proposed for isolates with an epidemiological link (12)] and case 5 (defined as the index case by field epidemiological investigation) which is separated from the cluster by a minimum number of 95 distinct SNPs (case 5 showed in Supplementary Table 1). Pairwise comparison of the WGSs from the isolates within the cluster revealed 20 SNPs (except case 5 showed in Table 4, all details of the different or the same mutation between each case are showed in the Supplementary File), 4 isolates are grouped around case 12 (defined as the super spreader) that mainly have single-, double-, or triple-SNP differences. Cases 2 and 4 with 7 SNP differences to case 12 shared 3 of the same SNP distances, indicating the miss of one strain node between them (Figure 2C).

**Table 4:** Single-nucleotide polymorphisms identified in the study (except case 5) by WGS.

**Category**	**Ref. position^a^**	**Gene**	**Base substitution**	**Classification**	**Change**
SNP used for phylogeny (*n* = 20)	335	dnaA	C → T	Non-synonymous	T200I
	19137	pbpA	A → G	Synonymous	
	907266	Rv0812	G → A	Non-synonymous	D282N
	1065308	Rv0954	C → T	Non-synonymous	T61I
	1192843	Rv1069c	C → T	Synonymous	
	1253425	Rv1129c	G → C	Non-synonymous	D370E
	1807087	chaA	T → G	Non-synonymous	S303A
	2538666		C → T	Intergenic	
	2604957	Rv2331A	A → T	Non-synonymous	K73M
	2729831	Rv2434c	G → T	Non-synonymous	P244T
	2729832	Rv2434c	G → C	Synonymous	
	2951317		C → T	Intergenic	
	2955447		G → A	Intergenic	
	2980915		C → T	Intergenic	
	3526043	nuoN	T → C	Non-synonymous	L85P
	3714256	sigJ	T → G	Non-synonymous	D26A
	3859116	Rv3439c	G → C	Non-synonymous	Q183E
	4206273	fadE36	G → A	Synonymous	
	4237927	dprE2	T → G	Non-synonymous	Stop255G
	4248143	embB	T → C	Synonymous	

a *Reference position relative to H37Rv genome*.

### Treatment Outcome of This Outbreak

All 22 cases with active pulmonary TB and 1 case with tuberculous pleurisy received standard treatment for tuberculosis with 2HRZE/4HR and were cured after 6 months of treatment. Until June 2021, after 3 years of the outbreak, our surveillance system did not detect TB in any of the people associated with the outbreak.

## Discussion

In this study, we described a TB outbreak among students in a boarding high school in Luoning, Henan, China. A total of 22 cases was diagnosed with active pulmonary TB and 1 case with tuberculous pleurisy, while no other cases were detected among teachers and household contacts. All cases, except case 23, detected in Grade-I were found in the same building which may share an epidemiological association. The infection may happen as early as January since case 7 left school for training during that time. We obtained the initial class of these active pulmonary cases before the science-humanities division, the cases were mainly distributed in the fourth floor of class B (n/N, 10/23). The occasional switching of classrooms based on exam scores increased the difficulty to source find. For example, case 13 has switched three times in half a year (from class-II B17 to B16 to B18, lived in N3-408 consistently). Unfortunately, the source of this outbreak remained unknown according to field epidemiological investigation.

LTBI is a state of persistent immune response to stimulation by *M. tuberculosis* antigens with no evidence of clinically manifested active TB. TST, which is based on cellular immune responses, is an available LTBI testing method (23). In our study, the TB infection rate here was lower; 311 cases (5.78%, 311/5,385) were tested LTBI with TST positive. Some research in schoolchildren and adolescents also observed lower than 10% positive rate of TB infection (24). LTBI testing and prophylactic treatment is facilitated to control TB, as the risk of development of active TB from LTBI is high. In our study, only 33 cases accepted a 3-month regimen of twice-weekly rifapentine plus isoniazid for LTBI treatment. Early identification of children with LTBI and timely administration of prophylactic treatment are important for TB control in children.

In 2017, 40,656 students reported having pulmonary TB in China, and a total of 835,193 cases were reported based on the national infectious disease network direct reporting system. The number of cases with students contributed to 4.78% of the total TB population, especially for high school students between the age of 16 and 18, which accounted for 42.7%. There were 21 outbreaks of TB in school detected based on the public health emergencies reporting system, among which 14 cases were reported in senior high schools in 2017. All events happened in boarding schools except for one case that was in non-boarding schools. To date, all 16 TB outbreaks among students occurred in boarding senior high schools in 2018.

We think there are several factors that contribute to the high-frequency cases happening in these boarding schools. First, delayed diagnosis of TB is commonly seen in schools with TB outbreaks (2, 3, 25). The median time from symptom onset to diagnosis of the index case was 43 days, with the longest being 258 days, and the shortest being 11 days in 2017. During the outbreak, delayed diagnosis (from 1 to 5 weeks) occurred with the index case and all other related cases. Because laboratory diagnostic approaches have low yield in teenagers (4, 26), the diagnosis of TB in this age group mainly relies on clinical manifestations (4). As shown in Table 2, the symptom of active pulmonary TB is inconspicuous because 13 students were asymptomatic. Only 4 cases (4/10) were found by active consultation among all the cases. Compared to the examination of fever (*P* = 0.010), cough and sputum analysis should be taken into more consideration as these manifestations are often ignored by individuals.

Second, the potential reason for this outbreak was the constant transmission of pathogens between class and dormitory as these students switch classes instead of dormitories. As shown in Table 1, 7 of the 8 cases found in class-II B16 were from other classes. As we know, students with lower test scores will be put into one class to improve their academic performance at school. To inhibit TB infection, teachers should focus on the health conditions of students who presented a sharp decline in academic performance. The limited space in the classroom and dormitory also creates conditions that contribute to the transmission of Mtb (27–29).

Finally, the lack of TB screen before school enrollment is another explanation for this outbreak. Although the outbreak was reported almost 2 years after the enrollment, we do not know when the infection began and how long the outbreak lasted (3, 30). Case 23 with asymptomatic manifestations in Grade-I revealed a cavitary TB image, a severe consequence may occur without screening. Compared to other countries with low TB incidence, the TB outbreak occurred more frequently among students in China (13, 25). Since 2017, TB screening of school students has been regulated by the National Health and Construction Commission in China. The policy will benefit the schools by focusing on TB prevention and control in China, although the policy implementation needs to be further promoted. A 20-year experience of TB prevention and control by health assessments of schools in Xinmi, Henan has proven the effectiveness of this measure.

TB cluster outbreaks occur occasionally in educational institutions in China. It is useful to analyze the transmission mechanism for tracing the source and to investigate the factors that obstruct the health care response to the break. A qualitative study in China for school TB incidents showed that the root of special illness experiences of school TB patients is the unique education system and sociocultural factors (25). The median time from symptom onset to diagnosis of the first case is 61 days, the longest is 296 days and the shortest is 5 days among all the 16 incidents that occurred in senior high schools in 2018. When students are sick, instead of focusing on disease treatment, they worry more about not being able to finish schoolwork and assignments on time due to the huge pressure from their families and high schools for the upcoming college entrance examination. The ignorance of schools and the level of local medical institutions become the biggest obstacles for TB diagnosis and treatment on time.

Conventional epidemiology is known as the gold standard for detection of recent transmission chains, but there are disadvantages especially in high TB incidence settings and is influenced by the method being used and the availability of the resources (12, 31). The fluke mind caused by the ignorance of school officials who tried to make their mishandling less serious by disguising a major incident as a minor one and then solve the issue quietly and quickly has also become a big obstacle to the field investigation in this case. Throughout the investigation, it was assumed that all the cases may be epidemiologically linked, as all of the cases were on the fourth floor except for case 2 being on the second floor within the same building. The index case 5 is likely belonging to the same clonal with the others. However, follow-up genome sequencing revealed this assumption to be false. SNP data suggested that case 5 shared a different transmission chain than the other cases.

Previous studies indicated that WGS sequencing provides a higher discrimination of clinical MTBC isolates compared to classical genotyping, e.g., based on MIRU-VNTR typing (15). Studies in China have shown that the proposed cut-off value of <12 SNPs could be used for identification of isolates involved in recent TB transmission (12, 32). In this case, the MST based on 15-focus MIRU-VNTR genotyping showed a similar chain of transmission defined by the SNP pattern, but the full level of genetic diversity within the same genotype of *M. tuberculosis* cannot be captured, which may comprise multiple distinct lineages (33).

Field epidemiological investigation combined with molecular epidemiology supports a complementary model to enhance the investigation of a tuberculosis outbreak (33). As shown in this outbreak, the index case in class-II B16 has a great epidemiologic association with other cases, but the student showed a more than 95 SNP distances with other isolates. Case 12, who was defined as the “super-spreader,” played a larger role of secondary transmission through person-to-person contact (33). After further investigation, we found that, except for case 5, all other cases did not stay in class-II B16 half a year before. This suggests it may be the time when the infection of Mtb occurred.

The cost of WGS has decreased and more sequencing analysis platforms have become convenient. Molecular epidemiology based on WGS, which can offer a higher resolution for MTBC outbreaks and better spatiotemporal correlation with the spread of the pathogen, will be widely used in TB prevalence and tracing analysis (10, 34–36). In summary, a performed health examination is essential to prevent the source of TB, improved efficiency of diagnosis of TB for the case and the primary case is necessary to reduce the risk for outbreaks, and timely and efficiently investigation and screening is fundamental to control TB transmission. Obviously, efforts should be taken to improve the living conditions in boarding schools, to educate students and teachers, and to provide training for school clinicians. The various impacts of control measures in different settings highlight the need for better preventive strategies and improved understanding of transmission at the population level. For 3 years after the TB outbreak, our surveillance system did not detect TB from any of the individuals associated with the outbreak. To public health practice, combined microbial genomic sequencing and epidemiologic approaches like we described in this study will become an important and tractable approach to tuberculosis control, especially in cluster investigation of TB outbreak in the academic setting.

## Data Availability Statement

The datasets presented in this study can be found in online repositories. The names of the repository/repositories and accession number(s) can be found below: NCBI PRJNA755247.

## Author Contributions

CL drafted the manuscript. HL and XO contributed to revising the manuscript. ML, DL, BZ, and JS participated in epidemiological investigation. YZ and GZ finalized the manuscript. All authors read and approved the final manuscript.

## Funding

This work was supported by Henan Province Medical Science and Technology Research Project: 2018020516.

## Conflict of Interest

The authors declare that the research was conducted in the absence of any commercial or financial relationships that could be construed as a potential conflict of interest.

## Publisher's Note

All claims expressed in this article are solely those of the authors and do not necessarily represent those of their affiliated organizations, or those of the publisher, the editors and the reviewers. Any product that may be evaluated in this article, or claim that may be made by its manufacturer, is not guaranteed or endorsed by the publisher.

## References

[1] World Health Organization. WHO 2018 Global Tuberculosis Report (2019).

[2] Fang Y Zhang L Tu C Ye D Fontaine R Ma H. Outbreak of pulmonary tuberculosis in a Chinese high school, 2009–2010. J Epidemiol. (2013) 23:307–12. 10.2188/jea.JE20120216 23774287 PMC3709544

[3] Chen W Xia Y Li X Zhou L Li C Wan K. A Tuberculosis outbreak among senior high school students in China in 2011. J Int Med Res. (2012) 40:1830–9. 10.1177/030006051204000521 23206464

[4] Ma MJ Yang Y Wang HB Zhu YF Fang LQ An XP. Transmissibility of tuberculosis among school contacts: an outbreak investigation in a boarding middle school, China. Infect Genet Evol. (2015) 32:148–55. 10.1016/j.meegid.2015.03.001 25757905 PMC4427569

[5] Wu X Pang Y Song Y Dong W Zhang T Wen S. Implications of a school outbreak of multidrug-resistant tuberculosis in Northern China. Epidemiol Infect. (2018) 146:584–8. 10.1017/S0950268817003120 29486815 PMC9134526

[6] Nikolayevskyy V Kranzer K Niemann S Drobniewski F. Whole genome sequencing of *Mycobacterium tuberculosis* for detection of recent transmission and tracing outbreaks: a systematic review. Tuberculosis. (2016) 98:77–85. 10.1016/j.tube.2016.02.009 27156621

[7] Hasnain SE O'Toole RF Grover S Ehtesham NZ. Whole genome sequencing: a new paradigm in the surveillance and control of human tuberculosis. Tuberculosis. (2015) 95:91–4. 10.1016/j.tube.2014.12.007 25586521

[8] van Embden J van Gorkom T Kremer K Jansen R van Der Zeijst B Schouls L. Genetic variation and evolutionary origin of the direct repeat locus of *Mycobacterium tuberculosis* complex bacteria. J Bacteriol. (2000) 182:2393–401. 10.1128/JB.182.9.2393-2401.2000 10762237 PMC111299

[9] Walker TM Monk P Smith EG Peto TE. Contact investigations for outbreaks of *Mycobacterium tuberculosis*: advances through whole genome sequencing. Clin Microbiol Infect. (2013) 19:796–802. 10.1111/1469-0691.12183 23432709

[10] Roetzer A Diel R Kohl TA Rückert C Nübel U Blom J. Whole genome sequencing versus traditional genotyping for investigation of a *Mycobacterium tuberculosis* outbreak: a longitudinal molecular epidemiological study. PLoS Med. (2013) 10:e1001387. 10.1371/journal.pmed.1001387 23424287 PMC3570532

[11] Ocheretina O Shen L Escuyer VE Mabou MM Royal-Mardi G Collins SE. Whole genome sequencing investigation of a tuberculosis outbreak in Port-au-Prince, Haiti Caused by a Strain with a “Low-Level” rpoB Mutation L511P - Insights into a Mechanism of Resistance Escalation. PLoS ONE. (2015) 10:e0129207. 10.1371/journal.pone.0129207 26039194 PMC4454571

[12] Luo T Yang C Peng Y Lu L Sun G Wu J. Whole-genome sequencing to detect recent transmission of *Mycobacterium tuberculosis* in settings with a high burden of tuberculosis. Tuberculosis. (2014) 94:434–40. 10.1016/j.tube.2014.04.005 24888866 PMC4409578

[13] Norheim G Seterelv S Arnesen TM Mengshoel AT Tonjum T Ronning JO. Tuberculosis outbreak in an educational institution in Norway. J Clin Microbiol. (2017) 55:1327–33. 10.1128/JCM.01152-16 28202795 PMC5405251

[14] Tyler AD Randell E Baikie M Antonation K Janella D Christianson S. Application of whole genome sequence analysis to the study of *Mycobacterium tuberculosis* in Nunavut, Canada. PLoS ONE. (2017) 12:e0185656. 10.1371/journal.pone.0185656 28982116 PMC5628838

[15] Wyllie DH Davidson JA Grace Smith E Rathod P Crook DW Peto TEA. A quantitative evaluation of MIRU-VNTR typing against whole-genome sequencing for identifying *Mycobacterium tuberculosis* transmission: a prospective observational cohort study. EBioMedicine. (2018) 34:122–30. 10.1016/j.ebiom.2018.07.019 30077721 PMC6116353

[16] Alcolea-Medina A Fernandez M Montiel N García M Sevilla C North N. An improved simple method for the identification of Mycobacteria by MALDI-TOF MS (Matrix-Assisted Laser Desorption- Ionization mass spectrometry). Sci Rep. (2019) 9:20216. 10.1038/s41598-019-56604-7 31882826 PMC6934676

[17] Zeng X Xu Y Zhou Y Li H Zheng R Tan Y. McSpoligotyping, a one-step melting curve analysis-based protocol for spoligotyping of *Mycobacterium tuberculosis*. J Clin Microbiol. (2018) 56:e00539–18. 10.1128/JCM.00539-18 29875194 PMC6062818

[18] Xia E Teo Y-Y Rong T-H. SpoTyping: fast and accurate in silico Mycobacterium spoligotyping from sequence reads. Genome Med. (2016) 8:19. 10.1186/s13073-016-0270-7 26883915 PMC4756441

[19] Supply P Allix C Lesjean S Cardoso-Oelemann M Rüsch-Gerdes S Willery E. Proposal for standardization of optimized mycobacterial interspersed repetitive unit-variable-number tandem repeat typing of *Mycobacterium tuberculosis*. J Clin Microbiol. (2006) 44:4498–510. 10.1128/JCM.01392-06 17005759 PMC1698431

[20] Amaro A Duarte E Amado A Ferronha H Botelho A. Comparison of three DNA extraction methods for Mycobacterium bovis, *Mycobacterium tuberculosis* and *Mycobacterium avium* subsp. avium. Lett Appl Microbiol. (2008) 47:8–11. 10.1111/j.1472-765X.2008.02372.x 18498320

[21] Mohammadi S Esfahani BN Moghim S Mirhendi H Zaniani FR Safaei HG. Optimal DNA isolation method for detection of nontuberculous mycobacteria by polymerase chain reaction. Adv Biomed Res. (2017) 6:133. 10.4103/2277-9175.217216 29279831 PMC5674650

[22] Cinquetti S Dalmanzio M Ros E Gentili D Ramigni M Grossi A. High rate of transmission in a pulmonary tuberculosis outbreak in a primary school, north-eastern Italy, 2019. Euro surveill. (2019) 24:1900332. 10.2807/1560-7917.ES.2019.24.24.1900332 31213222 PMC6582512

[23] Cui X Gao L Cao B. Management of latent tuberculosis infection in China: exploring solutions suitable for high-burden countries. Int J Infect Dis. (2020) 92S:S37–40. 10.1016/j.ijid.2020.02.034 32114201

[24] Gao L Li X Liu J Wang X Lu W Bai L. Incidence of active tuberculosis in individuals with latent tuberculosis infection in rural China: follow-up results of a population-based, multicentre, prospective cohort study. Lancet Infect Dis. (2017) 17:1053–61. 10.1016/S1473-3099(17)30402-4 28716677

[25] Zhang S Li X Zhang T Fan Y Li Y. The experiences of high school students with pulmonary tuberculosis in China: a qualitative study. BMC Infect Dis. (2016) 16:758. 10.1186/s12879-016-2077-y 27978819 PMC5159990

[26] Wootton SH Gonzalez BE Pawlak R Teeter LD Smith KC Musser JM. Epidemiology of pediatric tuberculosis using traditional and molecular techniques: Houston, Texas. Pediatrics. (2005) 116:1141–7. 10.1542/peds.2004-2701 16264001

[27] Richardson NL. Evaluating provider prescribing practices for the treatment of tuberculosis in Virginia, 1995 to 1998: an assessment of educational need. J Contin Educ Health Prof. (2000) 20:146–55. 10.1002/chp.1340200303 11232250

[28] Cohn DL. Treatment of latent tuberculosis infection: renewed opportunity for tuberculosis control. Clin Infect Dis. (2000) 31:120–4. 10.1086/313891 10913407

[29] Oelemann MC Fontes AN Pereira MA Bravin Y Silva G Degrave W. Typing of Mycobacterium tuberculosis strains isolated in Community Health Centers of Rio de Janeiro City, Brazil. Mem Inst Oswaldo Cruz. (2007) 102:455–62. 10.1590/S0074-02762007005000034 17612765

[30] Ilic M Spahic S Spahic M Spahic O Ilic I Tiodorovic B. Tuberculosis outbreak in a grammar school, Serbia, 2016. Ann Ist Super Sanita. (2019) 55:55–8. 10.4415/ANN_19_01_10 30968837

[31] Fox GJ Barry SE Britton WJ Marks GB. Contact investigation for tuberculosis: a systematic review and meta-analysis. Eur Respir J. (2013) 41:140–56. 10.1183/09031936.00070812 22936710 PMC3533588

[32] Liu Q Ma A Wei L Pang Y Wu B Luo T. China's tuberculosis epidemic stems from historical expansion of four strains of Mycobacterium tuberculosis. Nat Ecol Evol. (2018) 2:1982–92. 10.1038/s41559-018-0680-6 30397300 PMC6295914

[33] Gardy JL Johnston JC Ho Sui SJ Cook VJ Shah L Brodkin E. Whole-genome sequencing and social-network analysis of a tuberculosis outbreak. N Engl J Med. (2011) 364:730–9. 10.1056/NEJMoa1003176 21345102

[34] Bryant JM Schürch AC van Deutekom H Harris SR de Beer JL de Jager V. Inferring patient to patient transmission of *Mycobacterium tuberculosis* from whole genome sequencing data. BMC Infect Dis. (2013) 13:110. 10.1186/1471-2334-13-110 23446317 PMC3599118

[35] Zakham F Laurent S Esteves Carreira AL Corbaz A Bertelli C Masserey E. Whole-genome sequencing for rapid, reliable and routine investigation of transmission in local communities. New Microbes New Infect. (2019) 31:100582. 10.1016/j.nmni.2019.100582 31388433 PMC6669808

[36] Walker TM Ip CL Harrell RH Evans JT Kapatai G Dedicoat MJ. Whole-genome sequencing to delineate *Mycobacterium tuberculosis* outbreaks: a retrospective observational study. Lancet Infect Dis. (2013) 13:137–46. 10.1016/S1473-3099(12)70277-3 23158499 PMC3556524

