# Supplementary material for: Tuberculosis Outbreak in an Educational Institution in Henan Province, China
Source: Front Public Health. 2021 Oct 12;9:737488. doi: 10.3389/fpubh.2021.737488 (PMC8545879; doi:10.3389/fpubh.2021.737488)
Supplement: Supplementary file 2 [file Data_Sheet_2.docx]

**Supplementary Table. Single-nucleotide polymorphisms identified (case 5) by WGS**

| Category | Ref. position^1^ | Gene | Base substitution | Classification | Change |
| --- | --- | --- | --- | --- | --- |
| SNPs of case 5 | 27498 |  | G→A | Intergenic |  |
|  | 88362 | Rv0079 | C→T | synonymous |  |
|  | 166565 |  | A→G | Intergenic |  |
|  | 184393 | pntAa | A→G | non-synonymous | T772A |
|  | 208590 | Rv0177 | A→C | synonymous |  |
|  | 233346 |  | G→A | Intergenic |  |
|  | 250530 |  | C→A | Intergenic |  |
|  | 306213 | cobU | C→T | non-synonymous | R389Q |
|  | 311889 | Rv0260c | C→T | synonymous |  |
|  | 465070 | Rv0386 | G→A | non-synonymous | E1660K |
|  | 470033 | metZ | C→A | synonymous |  |
|  | 484005 | fadD30 | G→T | non-synonymous | R29L |
|  | 560466 | umaA | C→A | non-synonymous | F579L |
|  | 572436 | lprQ | T→G | non-synonymous | F727V |
|  | 638412 | Rv0547c | C→T | non-synonymous | G381S |
|  | 713345 | galK | G→A | synonymous |  |
|  | 739401 | mmaA1 | G→A | non-synonymous | H75Y |
|  | 854604 |  | C→T | Intergenic |  |
|  | 900903 |  | A→C | Intergenic |  |
|  | 922528 | Rv0830 | C→T | synonymous |  |
|  | 934784 | lpqR | C→T | non-synonymous | A65V |
|  | 941722 | Rv0845 | T→G | non-synonymous | I533S |
|  | 1130195 | ispE | C→T | non-synonymous | P5L |
|  | 1151304 | kdpD | C→A | non-synonymous | R2201L |
|  | 1164571 |  | A→G | Intergenic |  |
|  | 1181730 | fadD14 | G→A | synonymous |  |
|  | 1223898 | phoH2 | C→T | non-synonymous | P902L |
|  | 1225520 | Rv1097c | C→A | non-synonymous | G258C |
|  | 1265857 | Rv1138c | A→C | non-synonymous | L386R |
|  | 1266035 | Rv1138C | C→T | non-synonymous | V564I |
|  | 1271174 | Rv1144 | G→A | non-synonymous | V19I |
|  | 1309343 |  | G→A | Intergenic |  |
|  | 1368637 |  | C→T | Intergenic |  |
|  | 1404803 | Rv1257c | G→A | non-synonymous | L87F |
|  | 1409474 |  | G→T | Intergenic |  |
|  | 1440762 |  | T→C | Intergenic |  |
|  | 1446733 |  | G→A | Intergenic |  |
|  | 1494584 | glgP | T→G | non-synonymous | F21L |
|  | 1504335 | cysM | G→C | non-synonymous | E942D |
|  | 1523817 | moeY | C→T | synonymous |  |
|  | 1529136 |  | A→C | Intergenic |  |
|  | 1697552 |  | G→C | Intergenic |  |
|  | 1701229 |  | C→G | Intergenic |  |
|  | 1706657 |  | G→A | Intergenic |  |
|  | 1722228 | pks5 | A→T | non-synonymous | L146Q |
|  | 1827940 | cya | C→T | synonymous |  |
|  | 1851615 |  | T→C | Intergenic |  |
|  | 1875436 | pks7 | C→G | non-synonymous | R133G |
|  | 1980845 | Rv1751 | C→G | non-synonymous | Q1225E |
|  | 1998761 |  | T→G | Intergenic |  |
|  | 2092427 | guaB1 | G→A | synonymous |  |
|  | 2134071 | rpfC | A→G | non-synonymous | V341A |
|  | 2136612 | Rv1887 | A→G | non-synonymous | I355V |
|  | 2191959 | ephB | C→G | synonymous |  |
|  | 2204641 | Rv1961 | G→C | non-synonymous | A430P |
|  | 2204642 | Rv1961 | C→T | non-synonymous | A431V |
|  | 2216474 | mce3F | C→T | synonymous |  |
|  | 2392289 | mshC | G→A | synonymous |  |
|  | 2422061 |  | A→G | Intergenic |  |
|  | 2484251 | Rv2216 | T→C | non-synonymous | F626S |
|  | 2489315 |  | T→C | Intergenic |  |
|  | 2582569 |  | G→C | Intergenic |  |
|  | 2629847 | plcB | G→A | non-synonymous | T1067M |
|  | 2807336 | Rv2492 | A→G | non-synonymous | D59G |
|  | 2838897 | Rv2522c | C→G | non-synonymous | M769I |
|  | 2866169 | Rv2542 | T→G | non-synonymous | L1040R |
|  | 2878550 |  | G→A | Intergenic |  |
|  | 2888874 |  | G→A | Intergenic |  |
|  | 2904110 | dhaA | C→G | non-synonymous | P472A |
|  | 2923608 | ruvB | G→A | non-synonymous | A410V |
|  | 2951317 |  | C→T | Intergenic |  |
|  | 2965902 |  | T→C | Intergenic |  |
|  | 3056767 | Rv2743c | G→T | non-synonymous | P348T |
|  | 3264081 | ppsD | A→G | non-synonymous | T1834A |
|  | 3310174 | Rv2957 | G→A | synonymous |  |
|  | 3384209 | iscS | G→A | synonymous |  |
|  | 3472894 | prfB | T→C | synonymous |  |
|  | 3659168 | purK | A→C | non-synonymous | L534V |
|  | 3714256 |  | T→G | Intergenic |  |
|  | 3714377 |  | A→G | Intergenic |  |
|  | 3813266 | guaA | C→T | synonymous |  |
|  | 3819414 | Rv3401 | T→C | non-synonymous | V1373A |
|  | 3878472 | rpoA | G→A | synonymous |  |
|  | 3888283 | ilvB2 | G→T | non-synonymous | H1140N |
|  | 4042996 |  | C→A | Intergenic |  |
|  | 4046007 | Rv3603c | C→T | non-synonymous | V801M |
|  | 4062332 |  | C→T | Intergenic |  |
|  | 4114955 | Rv3673c | A→C | non-synonymous | L482W |
|  | 4153058 | ask | G→A | synonymous |  |
|  | 4202436 | proZ | G→A | synonymous |  |
|  | 4215433 | Rv3770c | C→T | non-synonymous | A234T |
|  | 4280254 | Rv3815c | G→T | non-synonymous | L222M |
|  | 4326340 |  | G→A | Intergenic |  |
|  | 4380822 | eccB2 | C→G | non-synonymous | M370I |
|  | 4381507 | eccB2 | G→T | non-synonymous | A1055E |

^1^Reference position relative to H37Rv genome.
